# Waist-height ratio and waist are the best estimators of visceral fat in type 1 diabetes

**DOI:** 10.1038/s41598-020-75667-5

**Published:** 2020-10-29

**Authors:** Erika B. Parente, Stefan Mutter, Valma Harjutsalo, Aila J. Ahola, Carol Forsblom, Per-Henrik Groop

**Affiliations:** 1 Folkhälsan Institute of Genetics, Folkhälsan Research Center, Biomedicum Helsinki, University of Helsinki, Helsinki, Finland; 2 Faculdade de Ciências Médicas da Santa Casa de São Paulo, São Paulo, Brazil; 3 Research Program for Clinical and Molecular Metabolism, Faculty of Medicine, University of Helsinki, Helsinki, Finland; 4 Abdominal Center, Nephrology, University of Helsinki and Helsinki University Hospital, Helsinki, Finland; 5 National Institute for Health and Welfare, Helsinki, Finland; 6 Department of Diabetes, Central Clinical School, Monash University, Melbourne, Australia

**Keywords:** Type 1 diabetes, Nephrology

## Abstract

Visceral fat is associated with cardiovascular and kidney disease. However, the relationship between body composition and anthropometric measures in type 1 diabetes is unknown. Using z-statistics, we ranked the ability of body mass index (BMI), waist circumference (WC), waist-hip ratio (WHR), waist-height ratio (WHtR) and a body shape index (ABSI) to capture measures of body composition from 603 Dual-energy-X-Ray-Absorptiometry scans of adults with type 1 diabetes. Albuminuria was defined as urinary albumin excretion rate of at least 30 mg/24 h. Women with albuminuria had higher visceral fat mass % (VFM%) (0.9 vs. 0.5%, p = 0.0017) and lower appendicular lean mass % (AppLM%) (25.4 vs 26.4%, p = 0.03) than those without. Men with albuminuria had higher VFM% (1.5 vs. 1.0%, p = 0.0013) and lower AppLM% (30.0 vs 32.3, p < 0.0001) than those without. In men, WHtR estimated VFM% best (z-statistics = 21.1), followed by WC (z = 19.6), BMI (z = 15.1), WHR (z = 14.6) and ABSI (z = 10.1). In women, the ranking was WC (z = 28.9), WHtR (z = 27.3), BMI (z = 20.5), WHR (z = 12.7) and ABSI (z = 10.5). Overall, the ranking was independent of albuminuria. Adults with type 1 diabetes and albuminuria have greater VFM% and lower AppLM% than those without. WHtR and WC best estimate the VFM% in this population, independently of albuminuria and sex.

## Introduction

The obesity epidemic is spreading worldwide. Along with this global trend, also people with type 1 diabetes have exhibited weight gain over the last years^1^. Such an increase in body weight will most likely have a harmful impact on the cardiovascular mortality rate of this population^2,3^. Previous research from our group showed that the mortality rate of individuals with type 1 diabetes increases starting from a body mass index (BMI) of 24.8 kg/m^2^, which is still within the normal range^1^. Therefore, it brings up the question of whether the BMI cut-off to define central obesity and cardiovascular risk in individuals with type 1 diabetes should be lower than the traditionally used 30 kg/m^2^.

BMI is the most commonly used tool to classify obesity, although it is an inadequate biomarker of abdominal obesity^4^. It does not differentiate muscle from fat nor give the precise information regarding the amount of body fat mass, which better defines obesity^5^ and which is positively associated with cardiovascular disease (CVD)^6,7^. Furthermore, BMI neither distinguishes women from men concerning their body fat nor provides any information about the fat distribution, which is relevant for the risk of CVD^8^. Given that obesity is causally related to diabetic nephropathy (DN) in individuals with type 1 diabetes^9^, it would be of utmost importance to understand how the anthropometric measures are related to their body composition and especially visceral fat, which has been associated with dyslipidemia, insulin resistance, CVD^8,10^, and chronic kidney disease^11,12^ in the general population.

The Dual-energy X-ray Absorptiometry (DXA) is a well-recognized method to study fat distribution and body composition^5^. Unfortunately, the high cost hinders its use in routine clinical practice. On the other side, anthropometric measures such as waist circumference (WC) and waist-hip ratio (WHR) are accessible and of low cost, although they have certain limitations^13^. Another practical anthropometric measure is the waist-height ratio (WHtR) that has been associated with CVD in the general population and has the advantage to have a unisex cut-off value of 0.5^13–16^, moreover, a meta-analysis has shown that WHtR is a better screening tool than WC and BMI for adult cardiometabolic risk factors^16^. A body shape index (ABSI) is another formula in which WC is adjusted for weight and height and has been a predictor of mortality, independently of BMI, in an American population from the National Health and Nutrition Examination Survey (NHANES)^17^.

Thus far, the relationship between these anthropometric measures and the body composition of adults with type 1 diabetes at different stages of DN, expressed as the presence or absence of albuminuria, has not previously been investigated. Therefore, this study aimed to explore these relationships and seek to identify the anthropometric measure to best estimate the visceral fat in this population with high cardiovascular risk.

## Results

Data were available from a total of 246 men (30.5% with albuminuria) and 357 women (20.4% with albuminuria) (Table 1). In both men and women, individuals with albuminuria were older, had longer diabetes duration, higher systolic blood pressure and worse glycaemic control (Table 1).

**Table 1:** Body composition and clinical characteristics of men and women with and without albuminuria.

	Men			Women		
	Normoalbuminuria	Albuminuria	p-value	Normoalbuminuria	Albuminuria	p-value
N	171	75		284	73	
Age (years)	44.3 (37.6, 52.9)	52.5 (41.3, 58.8)	0.0005	40.2 (32.5, 50.5)	49.8 (42.5, 56.1)	< 0.0001
Diabetes duration (years)	22.4 (18.9, 34.7)	39.3 (30.3, 43.8)	< 0.0001	24.0 (18.3, 34.7)	36.9 (24.8, 42.8)	< 0.0001
Age of diabetes onset (years)	18.5 (13.1, 25.8)	12.6 (7.5, 17.8)	0.0004	12.7 (8.3, 20.6)	12.2 (7.0, 19.2)	0.67
Systolic blood pressure (mmHg)	138 (129, 148)	145 (133, 160)	0.01	125 (116, 136)	135 (125, 150)	0.0002
Diastolic blood pressure (mmHg)	78 (72, 84)	76 (70, 82)	0.42	75 (69, 81)	77 (70, 84)	0.19
HbA1c (mmol/mol)	61 (54, 69)	66 (58, 74)	0.0072	61 (54, 70)	68 (59, 80)	0.006
HbA1c (%)	7.7 (7.1, 8.5)	8.2 (7.5, 8.9)	0.0056	7.7 (7.1, 8.6)	8.4 (7.5, 9.5)	0.0051
Total cholesterol (mmol/L)	4.49 (3.96, 5.08)	4.27 (3.60, 4.90)	0.20	4.47 (4.06, 5.03)	4.56 (3.92, 5.00)	0.34
HDL cholesterol (mmol/L)	1.42 (1.17, 1.75)	1.42 (1.17, 1.76)	0.96	1.63 (1.39, 1.91)	1.72 (1.39, 1.98)	0.15
Triglycerides (mmol/L)	1.00 (0.77, 1.38)	1.12 (0.87, 1.84)	0.17	0.82 (0.64, 1.11)	1.03 (0.75, 1.48)	0.0002
Body mass index (kg/m^2^)	25.5 (24.0, 27.5)	26.5 (24.4, 29.0)	0.09	25.4 (22.7, 28.5)	27.2 (23.1, 31.0)	0.0477
ABSI scaled (m^(11/6)^/kg^(2/3)^)	7.85 (7.55, 8.23)	8.21 (7.9, 8.56)	< 0.0001	7.37 (7.06, 7.75)	7.62 (7.34, 8.06)	0.0019
Height (cm)	181 (175, 185)	178 (173, 184)	0.12	166 (161, 170)	164 (160, 167)	0.29
Body weight (kg)	83.74 (75.56, 93.62)	83.84 (77.62, 93.24)	0.95	70.63 (62.19, 79.93)	72.41 (61.86, 84.86)	0.54
Waist circumference (cm)	93 (85, 99)	98 (89, 107)	0.01	81 (75, 91)	88 (78, 99)	0.0059
Hip circumference (cm)	99 (94, 103)	101 (95, 106)	0.19	99 (92, 107)	101 (93, 110)	0.44
Waist-hip ratio	0.93 (0.89, 0.98)	0.96 (0.93, 1.03)	0.0047	0.83 (0.79, 0.87)	0.85 (0.82, 0.90)	0.0068
Waist-height ratio	0.50 (0.48, 0.55)	0.55 (0.50, 0.59)	0.0002	0.49 (0.45, 0.55)	0.54 (0.48, 0.60)	0.0007
Body fat mass (kg)	21.87 (16.43, 27.97)	24.82 (21.06, 30.42)	0.0431	25.10 (18.77, 32.93)	28.72 (19.32, 35.25)	0.0398
Body fat mass percentage (%)	27.0 (21.5, 31.5)	29.1 (25.9, 33.3)	0.0331	35.6 (30.0, 41.2)	36.9 (32.0, 44.4)	0.39
Android fat mass (kg)	2.05 (1.28, 2.99)	2.64 (1.72, 3.48)	0.0090	1.86 (1.15, 2.70)	2.24 (1.13, 3.54)	0.07
Android fat mass percentage (%)	2.4 (1.6, 3.3)	3.0 (2.2, 3.9)	0.0295	2.7 (1.8, 3.5)	3.0 (1.9, 4.3)	0.16
Visceral fat mass (kg)	0.89 (0.46, 1.56)	1.30 (0.77, 2.05)	0.0070	0.39 (0.14, 0.80)	0.64 (0.26, 1.40)	0.0059
Visceral fat volume (cm^3^)	939.31 (482.67, 1652.63)	1376.19 (820.60, 2175.97)	0.0044	410.84 (150.89, 843.25)	681.71 (276.14, 1480.26)	0.0078
Visceral fat mass percentage (%)	1.0 (0.5, 1.7)	1.5 (0.9, 2.4)	0.0013	0.5 (0.2, 1.0)	0.9 (0.4, 1.7)	0.0017
Visceral-Android fat ratio	0.45 (0.33, 0.56)	0.53 (0.43, 0.68)	0.0066	0.22 (0.12, 0.31)	0.30 (0.19, 0.45)	0.0005
Body lean mass (kg)	58.63 (53.90, 65.34)	56.31 (53.00, 61.30)	0.06	42.84 (39.75, 46.25)	42.72 (38.37, 48.90)	0.87
Body lean mass percentage (%)	69.2 (65.0, 74.6)	67.3 (63.3, 70.4)	0.0436	61.0 (55.6, 66.1)	59.6 (52.8, 64.5)	0.29
Appendicular lean mass (kg)	27.02 (24.21, 30.35)	25.20 (22.66, 28.24)	0.0083	18.74 (17.10, 20.36)	18.35 (16.35, 21.21)	0.43
Appendicular lean mass percentage (%)	32.3 (30.2, 34.2)	30.0 (28.0, 31.4)	< 0.0001	26.4 (24.6, 28.6)	25.4 (23.0, 27.2)	0.0268

*HDL* High density lypoprotein, *HbA1c* glycated hemoglobin. Data shown in medians (interquartile ranges), *ABSI scaled* A body shape index scaled by 100. P-Values were calculated separately for men and women by permutation analysis with 10,000 permutations.

### Body composition according to the albuminuric stage

In men, BMI was no different between individuals with or without albuminuria (25.5 kg/m^2^ vs. 26.5 kg/m^2^, p = 0.08), albeit the two groups presented a different body composition. Men with albuminuria had greater BFM% (29.1% vs. 27.0%, p = 0.03), greater AFM% (3.0% vs. 2.4%, p = 0.02), and greater VFM% (1.5% vs. 1.0%, p = 0.001) compared with those without albuminuria (Table 1). On the other hand, men with albuminuria had lower BLM% (67.3% vs. 69.2%, p < 0.04) and lower AppLM% (30.0% vs. 32.3%, p < 0.0001) than the normoalbuminuric men (Table 1).

In women, BMI was greater in the albuminuric group (27.2 kg/m^2^ vs. 25.4 kg/m^2^, p = 0.04) than in the normoalbuminuric group. The BFM% (36.9% vs. 35.6%, p = 0.39) and the AFM% (3.0% vs. 2.7%, p = 0.16) were no different between women with or without albuminuria. However, we observed greater VFM% (0.9% vs. 0.5%, p = 0.001) in women with albuminuria (Table 1). Regarding the muscle mass, women with albuminuria had lower AppLM% (25.4% vs. 26.4%, p = 0.02) despite no difference in the BLM% (59.6% vs. 61.0%, p = 0.29) compared to the normoalbuminuric women (Table 1).

### Body composition according to sex

In the whole cohort (independently of the presence or absence of albuminuria), men and women had comparable BMI (25.9 kg/m^2^ vs. 25.9 kg/m^2^, respectively, p = 0.87), despite different body composition (Table 2). Men had lower BFM% (27.91% vs. 35.98%, p < 0.0001), greater BLM% (68.41% vs. 60.73%, p < 0.0001), and greater AppLM% (31.49% vs. 26.27%, p < 0.001) than women (Table 2). Although the AFM% was comparable between men and women (2.63% vs. 2.71%, p = 0.54), the VFM% was greater in men (1.20% vs. 0.58%, p < 0.0001).

**Table 2:** Body composition and clinical characteristics between men and women.

	Men	Women	p-*v*alue
N	246	357	
Age (years)	46.62 (38.13, 55.8)	41.75 (33.26, 52.9)	0.0008
Diabetes duration (years)	26.56 (20.24, 39.53)	26.42 (19.39, 37.57)	0.96
Age of diabetes onset (years)	16.40 (10.90, 23.17)	12.48 (8.19, 20.19)	< 0.0001
Systolic blood pressure (mmHg)	140.00 (129.00, 151.00)	127.00 (118.00, 140.00)	< 0.0001
Diastolic blood pressure (mmHg)	77.50 (71.00, 84.00)	75.00 (69.00, 82.00)	0.0303
HbA1c (mmol/mol)	62.00 (55.00, 70.00)	63.00 (54.00, 72.00)	0.86
HbA1c (%)	7.82 (7.18, 8.56)	7.92 (7.09, 8.74)	0.86
Total cholesterol (mmol/L)	4.42 (3.89, 5.06)	4.48 (4.04, 5.02)	0.44
HDL cholesterol (mmol/L)	1.42 (1.17, 1.75)	1.63 (1.39, 1.92)	< 0.0001
Triglycerides (mmol/L)	1.01 (0.78, 1.56)	0.84 (0.65, 1.21)	< 0.0001
Body mass index (kg/m^2^)	25.9 (24.1, 28.4)	25.9 (22.8, 29.1)	0.87
ABSI scaled (m^(11/6)^/kg^(2/3)^)	7.99 (7.65, 8.35)	7.42 (7.13, 7.8)	< 0.0001
Height (cm)	180.00 (174.85, 185.00)	165.50 (161.00, 170.00)	< 0.0001
Body weight (kg)	83.79 (77.13, 93.62)	70.98 (61.86, 80.70)	< 0.0001
Waist circumference (cm)	94.00 (86.00, 102.00)	82.00 (76.00, 93.00)	< 0.0001
Hip circumference (cm)	100.00 (95.00, 104.00)	100.00 (93.00, 108.00)	1.00
Waist-hip ratio	0.94 (0.89, 1.00)	0.83 (0.79, 0.87)	< 0.0001
Waist-height ratio	0.51 (0.48, 0.57)	0.5 (0.46, 0.56)	0.0384
Body fat mass (kg)	22.91 (17.40, 28.48)	25.39 (18.79, 33.44)	0.0228
Body fat mass percentage (%)	27.91 (22.69, 32.16)	35.98 (30.24, 41.61)	< 0.0001
Android fat mass (kg)	2.23 (1.36, 3.10)	1.90 (1.14, 2.94)	0.0143
Android fat mass percentage (%)	2.63 (1.86, 3.45)	2.71 (1.87, 3.63)	0.54
Visceral fat mass (kg)	1.00 (0.51, 1.73)	0.41 (0.16, 0.87)	< 0.0001
Visceral fat volume (cm^3^)	1063.96 (537.25, 1836.9)	438.67 (170.57, 926.56)	< 0.0001
Visceral fat percentage (%)	1.20 (0.64, 1.93)	0.58 (0.26, 1.10)	< 0.0001
Visceral-Android fat ratio	0.48 (0.37, 0.61)	0.23 (0.13, 0.33)	< 0.0001
Body lean mass (kg)	57.9 (53.54, 64.52)	42.83 (39.66, 46.3)	< 0.0001
Body lean mass percentage (%)	68.41 (64.19, 73.26)	60.73 (55.45, 66.04)	< 0.0001
Appendicular lean mass (kg)	26.42 (23.64, 29.67)	18.62 (16.94, 20.44)	< 0.0001
Appendicular lean mass percentage (%)	31.49 (29.41, 33.47)	26.27 (24.34, 28.43)	< 0.0001
Normoalbuminuria (%)	69.51	79.55	0.0050
Microalbuminuria (%)	11.38	12.32	0.79
Macroalbuminuria (%)	19.11	8.12	0.0001
CKD stage 1 (%)	67.48	70.31	0.46
CKD stage 2 (%)	20.33	22.41	0.54
CKD stage 3 (%)	6.50	4.48	0.35
CKD stage 4 (%)	1.63	1.40	1.00
CKD stage 5 (%)	4.07	1.40	0.05

*HDL* High density lypoprotein, *HbA1c* glycated hemoglobin, *CKD* Chronic kidney disease. Data shown in medians (interquartile ranges), *ABSI scaled* A body shape index scaled by 100. p-values were calculated by permutation analysis with 10,000 permutations.

### Associations between body composition and anthropometric measures

#### Measures of BFM%

WHtR, followed by the WC, was the anthropometric measure to best estimate the BFM% in men, independently of albuminuria (Table 3). In women, BMI followed by WC was the measure to best estimate the BFM%, also independently of albuminuria (Table 3). WHtR explained 60% of the BFM% variation in all men and 63% of the BFM% variation in all women. The BMI explained 47% of the BFM% variation in all men and 70% of the BFM% variation in all women (Table 3). In our linear model, the WHtR cut-off of 0.5 corresponded to a BFM% of 25.0 in all men and a BFM% of 34.6 in all women (Table 4).

**Table 3:** Associations between body composition and anthropometric measures according to the albuminuric stages.

Dependent variable	Independent variable	MEN				WOMEN			
		beta value	p value	r^2^	zeta value	beta value	p value	r^2^	zeta value
**ALL**									
BFM%	BMI	1.27 [1.10, 1.45]	5.75E−35	0.47	14.5689	1.30 [1.21, 1.39]	2.62E−94	0.70	28.6701
BFM%	WAIST	0.46[0.41, 0.50]	3.73E−47	0.58	18.1633	0.49 [0.45, 0.53]	2.19 E−79	0.63	24.7941
BFM%	WHR scaled	5.84 [4.78, 6.89]	1.01E−22	0.33	10.8811	3.58 [2.39, 4.76]	6.79E−09	0.09	5.9407
BFM%	WHtR scaled	8.53 [7.65, 9.41]	4.19E−50	0.60	19.0456	7.94 [7.29, 8.58]	2.31E−77	0.63	24.2819
BFM%	ABSI scaled	4.49 [3.53, 5.46]	2.24E−17	0.26	9.1591	1.98 [1.01, 2.96]	7.53E−05	0.04	4.006
VFM%	BMI	0.17 [0.15, 0.19]	1.23E−36	0.53	15.0568	0.10 [0.09, 0.11]	3.26E−62	0.56	20.5214
VFM%	WAIST	0.06 [0.05, 0.06]	5.16E−52	0.65	19.6216	0.05 [0.04, 0.05]	4.98E−95	0.71	28.8623
VFM%	WHR scaled	0.91 [0.79, 1.03]	3.91E−35	0.51	14.6141	0.58 [0.49, 0.67]	6.93E−31	0.34	12.7462
VFM%	WHtR scaled	1.15 [1.05, 1.26]	9.52E−57	0.68	21.0667	0.73 [0.67, 0.78]	5.66E−89	0.69	27.2620
VFM%	ABSI scaled	0.63 [0.50, 0.75]	3.04E−20	0.36	10.097	0.41 [0.33, 0.48]	9.06E−23	0.27	10.539
BLM%	BMI	− 1.20 [− 1.36, −1.03]	8.21E−34	0.46	− 14.2243	− 1.22 [− 1.31, −1.14]	1.11E−91	0.69	− 27.9460
BLM%	WAIST	− 0.43 [− 0.48, −0.38]	1.08E−45	0.57	− 17.7289	− 0.46 [− 0.50, −0.43]	3.01E−73	0.63	− 24.4874
BLM%	WHR scaled	− 5.48 [− 6.49, −4.47]	4.39E−22	0.33	− 10.6808	− 3.38 [− 4.50, −2.26]	7.87E−08	0.09	− 5.9310
BLM%	WHtR scaled	− 8.02 [− 8.87, −7.17]	2.49E−48	0.59	− 18.5140	− 7.47 [− 8.09, −6.86]	1.10E−69	0.62	− 23.9328
BLM%	ABSI scaled	− 4.24 [− 5.16, −3.32]	4.47E−17	0.26	− 9.0586	− 1.91 [− 2.83, −0.99]	5.52E−05	0.05	− 4.0823
**NORMOALBUMINURIA**									
BFM%	BMI	1.29 [1.07, 1.51]	2.75E−23	0.45	11.6211	1.33 [1.22, 1.44]	3.62E−72	0.68	24.6341
BFM%	WAIST	0.47 [0.40, 0.53]	1.37E−31	0.56	14.5603	0.51 [0.47, 0.56]	2.79E−59	0.61	20.9316
BFM%	WHR scaled	5.50 [4.15, 6.86]	1.74E−13	0.28	8.0184	2.90 [1.51, 4.28]	5.11E−05	0.06	4.1141
BFM%	WHtR scaled	9.01 [7.85, 10.17]	7.53E−34	0.59	15.3684	8.18 [7.40, 8.97]	7.16E−58	0.60	20.5365
BFM%	ABSI scaled	4.25 [3.09, 5.41]	1.73E−11	0.24	7.219	1.38 [0.33, 2.43]	9.96E−03	0.02	2.5948
VFM%	BMI	0.15 [0.13, 0.18]	1.55E−24	0.49	12.0640	0.09 [0.08, 0.10]	4.86E−49	0.55	18.0909
VFM%	WAIST	0.06 [0.05, 0.06]	1.28E−36	0.63	16.3685	0.04 [0.04, 0.04]	8.21E−73	0.69	24.8252
VFM%	WHR scaled	0.83 [0.69, 0.96]	4.22E−24	0.49	11.9099	0.44 [0.34, 0.53]	1.31E−17	0.25	9.1377
VFM%	WHtR scaled	1.11 [0.99, 1.23]	1.62E−40	0.67	17.8027	0.65 [0.60, 0.70]	1.21E−69	0.68	23.8945
VFM%	ABSI scaled	0.56 [0.43, 0.69]	8.10E−15	0.34	8.5327	0.30 [0.22, 0.37]	1.61E−14	0.21	8.1069
BLM%	BMI	− 1.21 [− 1.342 −1.00]	2.31E−22	0.44	− 11.2931	− 1.25 [− 1.35, −1.15]	9.06E−70	0.67	− 23.9314
BLM%	WAIST	− 0.44 [− 0.50, −0.38]	1.80E−30	0.55	− 14.1632	− 0.49 [− 0.53, −0.44]	2.18E−58	0.60	− 20.6812
BLM%	WHR scaled	− 5.17 [− 6.46, −3.87]	4.19E−13	0.27	− 7.8683	− 2.73 [− 4.05, −1.42]	5.68E−05	0.06	− 4.0881
BLM%	WHtR scaled	− 8.47 [− 9.59, −7.35]	1.32E−32	0.57	− 14.9230	− 7.71 [− 8.46, −6.96]	1.15E−56	0.59	− 20.1996
BLM%	ABSI scaled	− 4.02 [− 5.13, −2.91]	2.23E−11	0.24	− 7.1732	− 1.36 [− 2.35, −0.36]	7.62E−03	0.03	− 2.6878
**ALBUMINURIA**									
BFM%	BMI	1.19 [0.91, 1.46]	7.13E−13	0.51	8.7138	1.23 [1.06, 1.40]	1.06E−22	0.75	14.4528
BFM%	WAIST	0.42 [0.34, 0.50]	9.42E−16	0.59	10.2710	0.45 [0.39, 0.52]	1.17E−21	0.73	13.8017
BFM%	WHR scaled	6.42 [4.59, 8.25]	1.21E−09	0.40	6.9794	5.76 [3.31, 8.21]	1.32E−05	0.24	4.6882
BFM%	WHtR scaled	7.60 [6.17, 9.03]	2.36E−16	0.61	10.6015	7.62 [6.49, 8.75]	4.90E−21	0.72	13.4210
BFM%	ABSI scaled	4.23 [2.44, 6.01]	1.14E−05	0.24	4.7174	3.93 [1.74, 6.13]	6.41E−04	0.16	3.5746
VFM%	BMI	0.19 [0.15, 0.23]	9.37E−14	0.60	9.1872	0.13 [0.10, 0.15]	3.69E−15	0.60	10.0194
VFM%	WAIST	0.07 [0.05, 0.08]	7.43E−16	0.65	10.3277	0.05 [0.05, 0.06]	2.46E−22	0.75	14.2240
VFM%	WHR scaled	1.07 [0.80, 1.34]	2.01E−11	0.54	7.9376	0.97 [0.74, 1.20]	2.49E−12	0.52	8.4698
VFM%	WHtR scaled	1.19 [0.97, 1.41]	7.48E−17	0.67	10.8776	0.87 [0.73, 1.01]	5.88E−20	0.71	12.7699
VFM%	ABSI scaled	0.63 [0.35, 0.91]	2.74E−05	0.32	4.4807	0.72 [0.50, 0.94]	6.42E−09	0.40	6.608
BLM%	BMI	− 1.12 [− 1.38, −0.86]	1.23E−12	0.51	− 8.5875	− 1.15 [− 1.31, −0.99]	1.75E−22	0.75	− 14.3175
BLM%	WAIST	− 0.40 [− 0.48, −0.32]	2.09E−15	0.59	− 10.0821	− 0.42 [− 0.48, −0.36]	2.06E−21	0.73	− 13.6507
BLM%	WHR scaled	− 6.03 [− 7.79, −4.27]	2.18E−09	0.39	− 6.8395	− 5.45 [− 7.76, −3.15]	1.15E−05	0.25	− 4.7251
BLM%	WHtR scaled	− 7.15 [− 8.53, −5.77]	8.14E−16	0.60	− 10.3058	− 7.16 [− 8.23, −6.09]	6.63E−21	0.72	− 13.3407
BLM%	ABSI scaled	− 3.94 [− 5.65, −2.23]	1.81E−05	0.23	− 4.593	− 3.68 [− 5.75, −1.61]	6.91E−04	0.16	− 3.5513

*BMI* body mass index (kg/m^2^), *WAIST* waist circumference (cm), *WHR scaled* waist-hip ratio*10, *WHtR scaled* waist-height ratio*10, *ABSI scaled* a body shape index scaled by standard deviation, *BFM %* body fat mass percentage, *BLM %* body lean mass percentage, *VFM %* visceral fat mass percentage, *Beta* linear regression coefficients adjusted for age, *r*^*2*^ coefficient of determination from the linear regression, the relevance ranking of each variable was based on the z statistics.

**Table 4:** Percentage of total and visceral body fat related to BMI, WC and WHtR cut-offs according to the albuminuric stages.

Sex	BMI 25	BMI 30	WC (94/80)	WHtR 0.5
**BFM%**				
All				
Men	25.6	31.8	26.9	25.0
Women	33.9	40.4	33.3	34.6
Normoalbuminuria				
Men	25.0	31.2	26.7	24.9
Women	34.0	40.6	33.4	34.9
Albuminuria				
Men	27.2	33.0	27.4	25.4
Women	33.9	40.0	32.7	33.5
**VFM%**				
All				
Men	1.2	2.0	1.3	1.1
Women	0.6	1.1	0.5	0.7
Normoalbuminuria				
Men	1.1	1.8	1.3	1.0
Women	0.6	1.0	0.5	0.6
Albuminuria				
Men	1.5	2.3	1.5	1.1
Women	0.8	1.5	0.6	0.7

*BMI* body mass index (kg/m^2^), *WC* waist circumference (cm), *WHtR* waist-height ratio, *BFM%* body fat mass percentage, *VFM%* visceral fat mass percentage. WC was considered normal if < 94 cm for men and < 80 cm for women. WHtR was considered normal if < 0.5 for both sexes. BMI was considered normal if < 25 kg/m^2^, overweight if ≥ 25 and < 30 kg/m^2^ and obese if ≥ 30 kg/m^2^.

#### Measures of VFM%

**Figure 1:**
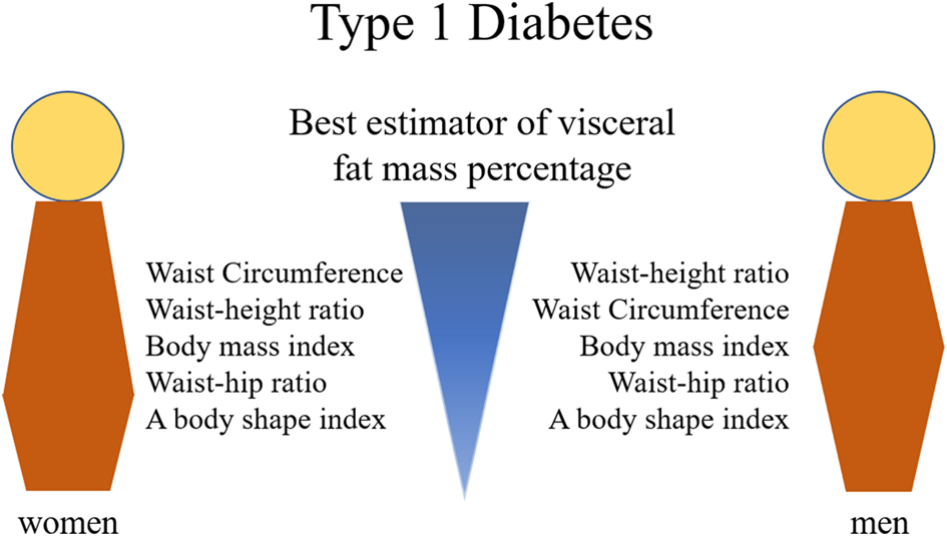
Associations between visceral fat and anthropometric measures in adults with type 1 diabetes according to sex.

WHtR, followed by the WC, was the anthropometric measure to best estimate the VFM% in men, while WC followed by WHtR was the best in women, independently of albuminuria (Fig. 1 and Table 3). The WHtR explained 68% of the VFM% variation in all men and 69% of the VFM% variation in all women (Table 3). WC explained 65% of the VFM% variation in all men and 71% of the VFM% variation in all women (Table 3). In our linear model, the WHtR cut-off of 0.5 corresponded to a VFM% of 1.1 in all men and a VFM% of 0.7 in all women (Table 4).

#### Measures of BLM%

WHtR, followed by WC, was the anthropometric measure to best estimate BLM% in all men, while BMI followed by WC was the best measure in women, independently of albuminuria (Table 3). The WHtR explained 59% of the BLM% variation in all men and 62% of the BLM% variation in all women (Table 3). BMI explained only 46% of the BLM% variation in all men but 69% of the BLM% variation in all women (Table 3).

The BLM% was negatively associated with the VFM% in all men (beta − 5.58 [− 6.17, − 4.98], r2 = 0.59, p = 7.09 × 10^–48^) and in all women (beta − 7.88 [− 8.65, − 7.11], r^2^ = 0.53, p = 1.23 × 10^–60^). The same association pattern was seen for both the normoalbuminuric and the albuminuric stage.

The BFM%, VFM% and BLM% associations with anthropometric measures according to the albuminuric stages are shown in Table 3.

WHR and ABSI showed a low association with the BFM%, VFM% and BLM%, independently of the albuminuric stage and sex (Table 3).

### Misclassification of body fat

Considering the normal threshold of BFM% below 25 for men and below 30 for women, the BMI misclassified 26.4% of the total cases (Table 5) by underestimating the body fat percentage in 20.6% of the cases and overestimating it in 5.8% (Table 5). The misclassification of BFM% by WC, WHR and WHtR were 22.6% (Table S1), 37.1% (Table S2) and 26.6% (Table S3), respectively.

**Table 5:** Misclassification of body fat percentage by BMI.

	BMI < 25 (n)	BMI ≥ 25 (n)	TOTAL (n)
Normal body fat percentage (n, %)	137 (22.7)	35 (5.8)	172 (28.5)
Excess body fat percentage (n, %)	124 (20.6)	307 (50.9)	431 (71.5)
Number (%) of DXAs	261 (43.3)	342 (56.7)	603 (100.00)

The normal body fat percentage was considered: ≤ 25 for men and ≤ 30 for women. DXA: Dual-energy X-Ray Absorptiometry. Percentages are based on the total number of 603 scans.

## Discussion

In this study, we investigated for the first time the body composition of individuals with type 1 diabetes with and without albuminuria. We showed that individuals with albuminuria, regardless of sex, had greater visceral fat percentage and lower appendicular lean mass percentage, which is a dangerous combination regarding the risk of CVD^8,18,19^. This finding is even more relevant if we consider that this population already has a high cardiovascular risk because of diabetes and DN^1,3,20^.

We showed that individuals with similar BMI have very different body composition, especially concerning the percentage of visceral fat and lean mass. This result emphasizes the importance of knowing the body composition instead of only BMI in a high cardiovascular risk population. The increase in body weight with a central distribution favours the accumulation of visceral fat that leads to an inflammatory state and insulin resistance^10^, which has been associated with kidney disease^11,12,21^. To make the situation worse, low lean mass^22^ and kidney disease^23^ are also associated with muscle insulin resistance. Considering that this is a cross-sectional study, it is not possible to say if the visceral fat, which is linked to chronic inflammation and insulin resistance, is contributing to the development of albuminuria or the albuminuric stage is worsening the visceral fat and insulin resistance due to physical inactivity, inflammation, changes in the microbiome or other factors^21^.

Most importantly, in this study, we found a strong association between VFM% and simple measures such as WHtR and WC, independently of the albuminuric stage and sex. We are not aware of any other study in individuals with type 1 diabetes that have previously assessed such relationships, especially looking at different stages of albuminuria. WHtR has been associated with central obesity and cardiovascular risk in the general population and in people with type 2 diabetes^13–15^. Furthermore, in a large prospective study including 109,536 postmenopausal women, it has been linked to cardiovascular events^24^. However, the relationship between WHtR and VFM% has never been described in individuals with type 1 diabetes with and without albuminuria. Given that DN increases cardiovascular mortality several-fold^20,25^ and that visceral fat is closely associated with CVD^8, 18^, our results regarding the association between WHtR and visceral fat are consistent with the literature which has shown the WHtR is a better screening tool than BMI for cardiometabolic risk factors^16^. Therefore, this study brings up new important information regarding central obesity in individuals with type 1 diabetes, a subject that most of the time has been related to type 2 diabetes. In this respect, it is important to acknowledge that obesity is increasing among individuals with type 1 diabetes and at the same time, there has been an increase in the mortality rate starting from a normal range of BMI^1^.

Another novel finding of this study was the negative association between BLM% and WHtR and WC, independently of the albuminuric stage. Although BLM% was best estimated by WHtR in men and by BMI in women, the simple measurement of WC was the second-best for the estimation of BLM% in both sexes. A plausible explanation for why WC and WHtR can estimate the percentage of body lean mass is the negative association between BLM% and VFM%. Although a recent publication showed that the fat-free mass was not associated with CVD^7^, it does not exclude the relevance of our findings, since one has to take into consideration that the fat-free mass measured by bioimpedance includes not only the muscle mass and, in our study, we measured the body lean mass by DXA, which has better accuracy than the bioimpedance^5^. From a clinical perspective, we found a simple and accessible tool to estimate the body lean mass in individuals with type 1 diabetes, independently of albuminuria.

Since low skeletal muscle mass is linked to CVD^19^ and muscle wasting has been associated with premature death in individuals with end-stage renal disease^26^ another important clinical finding was that, independently of sex, individuals with albuminuria have lower AppLM% compared to those without albuminuria, which might contribute to the increase in the cardiovascular risk of this high-risk population. Muscle wasting is not rare in individuals with end-stage renal disease^26,27^ and although we did not include such individuals in our analyses, we showed that individuals with type 1 diabetes at the earlier stages of DN (micro and macroalbuminuria) already show a decrease in their BLM% compared to those with normoalbuminuria.

According to previous publications including individuals with obesity and/or type 2 diabetes, in the current study, the ABSI was positively associated with central obesity and negatively associated with body lean mass^28,29^. However, it was inferior to the other anthropometric measures for the estimation of BFM%, VFM% and BLM% in our sample composed by Caucasian-Finnish individuals with type 1 diabetes. Since ABSI is a formula composed by WC adjusted for weight and height, the association between ABSI and body composition may vary depending on the characteristics of the studied population and on ethnicity.

In our study, BMI was not the anthropometric measure to best estimate VFM% in both sexes, and this inability of BMI to reflect the abdominal fat has been discussed erlier^4^. It was not useful to estimate BFM% and BLM% in men either, although it was in women. The relationship between BMI and BFM% was studied previously in the general population^30^ and the percentage of body fat mass related to the BMI was similar to our study. However, the American study^30^ did not investigate the associations between VFM%, BLM% with BMI, not either with WHtR and WC such as our study.

Furthermore, BMI misclassified BFM% in 26% of the total cases and underestimated it in 21% of them. Although the level of misclassification by BMI in our study is lower than in a previous study^31^, it might be explained by the different methods used to assess the body composition. In the previous study, they used bioimpedance while we used DXA, which provides better accuracy^5^. However, such as misclassification is clinically relevant, since individuals considered to have normal body weight by BMI might, in fact, have an excess of body fat and visceral fat, which are both closely associated with cardiovascular mortality^8,13,32,33^. The misclassification by BMI is another possible explanation of why the mortality rate in individuals with type 1 diabetes starts to increase already from the normal range of BMI^1^ Interestingly, the WC and WHtR misclassify the BFM% at least similarly to BMI, although they are in fact measures to estimate the central fat and not the total body fat. This finding is clinically important since a simple measure of WHtR or WC could not only better estimate visceral fat than BMI, but was able to classify obesity (BFM%) as well as BMI.

Another novelty of this study is to show, by our linear models, how much of the body fat mass and visceral fat mass percentages are related to the cut-offs of BMI, WC and WHtR. Interestingly, the BFM% and VFM% related to the BMI of 25 kg/m^2^ are similar to BFM% and VFM% related to the WHtR of 0.5. Therefore, our finding may provide a clue, why there is an increase in the mortality rate of this population starting from a BMI of 24.8 kg/m^2^ ^1^. These results may question whether the BMI of 25 kg/m^2^ is the best cut-off to define central obesity and cardiovascular risk in individuals with type 1 diabetes.

A limitation of this study is that we can not exclude confounding factors such as lifestyle and ethnicity. Since we studied a homogenous all-Caucasian Finnish population with type 1 diabetes and there are different thresholds for waist circumference and BMI for different ethnicities^4,34^, our results may not be applicable for all ethnicities. Another limitation is its cross-sectional design; therefore, it is not possible to conclude any causality between the associations we found nor any prediction of CVD risk. However, these results motivate further prospective studies to investigate the impact of body composition on chronic diabetes complications in individuals with type 1 diabetes. Another strength is its wide applicability to clinical practice since we here provide easily applicable tools to estimate the percentage of visceral fat and lean mass in a population with a high cardiovascular risk.

In conclusion, this study shows that simple measures such as WHtR and WC can estimate the VFM% in adults with type 1 diabetes independently of albuminuric stage and sex. Furthermore, it showed that individuals with type 1 diabetes and albuminuria, a population of high CVD risk, have greater VFM% and lower AppLM% compared to those with normoalbuminuria. From the clinical perspective, this study supports the routine monitoring of WHtR in adults with type 1 diabetes.

## Methods

### Study design and population

In this cross-sectional study, we explored the body composition and its relationship with anthropometric measures in individuals with type 1 diabetes with and without albuminuria. Adults with type 1 diabetes (n = 579) from the Finnish Diabetic Nephropathy (FinnDiane) Study cohort that had a DXA scan (n = 603) between August 2011 and June 2019 were included in this analysis. For 24 individuals, two scans were performed 5.3 ± 1.4 years apart as part of a regular follow-up visit. Sensitivity analysis including only the first scan (n = 579) for each individual led to the same conclusions (data not shown). Since 1997 the FinnDiane Study is recruiting and thoroughly characterizing individuals with type 1 diabetes 18 years or older at 93 centers across Finland. Type 1 diabetes is defined as age at onset of diabetes under 40 years and permanent insulin treatment initiated within a year from the diabetes diagnosis. The study protocol is designed according to the principles of the Declaration of Helsinki as revised in 2000 and was approved by the Ethics Committee of Helsinki and Uusimaa Hospital District. Written informed consents are obtained from each FinnDiane Study participant.

### Diabetic nephropathy (DN) stage

Normoalbuminuria was defined as an urinary albumin excretion rate (UAER) < 20 µg/min or < 30 mg/24 h in at least two out of three urine samples. Microalbuminuria was defined as a UAER ≥ 20 and < 200 µg/min or ≥ 30 and < 300 mg/24 h and macroalbuminuria as a UAER ≥ 200 µg/min or ≥ 300 mg/24 h. Individuals with micro- or macroalbuminuria were pooled together for analyses and will be referred throughout the paper as the albuminuric group. Individuals with end-stage renal disease (either on dialysis or have received a kidney transplant) were not included in this study.

### Body composition and anthropometric measurements

Body composition was evaluated by DXA (GE Healthcare Lunar version 16, Wisconsin, USA) according to the manufacturer’s instructions and visceral fat was measured by CoreScan^35^. A total body fat percentage below 25 was considered normal for men and below 30 for women^30,31^. The body fat mass percentage (BFM%), android fat mass percentage (AFM%), visceral fat mass percentage (VFM%), body lean mass percentage (BLM%), and appendicular lean mass percentage (AppLM%) were calculated by dividing the variable (grams) by total body weight (grams). Appendicular lean mass refers to the lean mass of both legs and arms.

BMI was calculated as total body weight (kilograms) divided by the square of the height (meters)^36^ and individuals were stratified by their BMI as normal weight (19–24.9 kg/m^2^), overweight (25–29.9 kg/m^2^) and obese (≥ 30 kg/m^2^)^36^. WC was measured in centimeters by a stretch‐resistant tape at the horizontal plane midway in the distance of superior iliac crest and the lower margin of the last rib^34^. The WC was considered normal if it was below 94 cm for men and below 80 cm for women^37^. The hip circumference was measured with the same tape around the widest part over the great trochanters and WHR was calculated by dividing the WC by the hip circumference^34^. A WHR below 0.9 for men and below 0.85 for women were considered normal^34^. The WHtR was calculated by dividing the WC by the height and values below 0.5 were considered normal for both men and women^13^. ABSI was calculated by the formula WC/(BMI^2/3^height^½^)^17^.

### Statistical analyses

For the analyses, we split the cohort into men and women and further distinguished between individuals with and without albuminuria.

Data on categorical variables are presented as frequencies, while continuous variables as medians (interquartile ranges, IQR). Between-group comparisons were conducted via permutation tests with 10,000 permutations. To compare the anthropometric measures for their capacity to estimate body composition, we performed linear regression analyses adjusted for age with the measurements of body composition as the dependent variables and anthropometric measures as the independent variables. The regressions’ R^2^ values were used to describe the proportion of the variance of a body composition measure that is explained by an anthropometric measure. The relevance ranking of each variable was based on the z statistics allowing comparisons across risk factors independently of their measurement units^38^. WHR and WHtR were scaled by a factor of 10. All analyses were performed in R^39^.

## Supplementary information

Supplementary Information.

## Abbreviations

AFM: Android fat mass
AFM%: AFM/total body weight × 100
AppLM: Appendicular-lean-mass (both legs and arms lean mass)
AppLM%: AppLM/total body-weight × 100
BFM: Body fat mass
BFM%: BFM/total body weight × 100
BLM: Body lean mass
BLM%: BLM/total body weight × 100
BMI: Body mass index
DN: Diabetic nephropathy
DXA: Dual-energy X-ray Absorptiometry
HDL: High-density lipoprotein cholesterol
VFM: Visceral fat mass
VFM%: VFM/total body weight × 100
WC: Waist circumference
WHR: Waist-hip-ratio
WHtR: Waist-height ratio

## References

[1] Dahlström EH (2019). Body mass index and mortality in individuals with type 1 diabetes. J. Clin. Endocrinol. Metab..

[2] Conway B (2009). Adiposity and mortality in type 1 diabetes. Int. J. Obes..

[3] Edqvist J (2019). BMI, mortality, and cardiovascular outcomes in type 1 diabetes: findings against an obesity paradox. Diabetes Care.

[4] Ross R (2020). Waist circumference as a vital sign in clinical practice: a Consensus Statement from the IAS and ICCR Working Group on Visceral Obesity. Nat. Rev. Endocrinol..

[5] Parente EB (2016). Is body mass index still a good tool for obesity evaluation?. Arch. Endocrinol. Metab..

[6] Larsson SC, Bäck M, Rees JMB, Mason AM, Burgess S (2020). Body mass index and body composition in relation to 14 cardiovascular conditions in UK Biobank: a Mendelian randomization study. Eur. Heart J..

[7] Larsson SC, Burgess S (2020). Fat mass and fat-free mass in relation to cardiometabolic diseases: a two-sample Mendelian randomization study. J. Intern. Med..

[8] Després J-P (2012). Body fat distribution and risk of cardiovascular disease. Circulation.

[9] Todd JN (2015). Genetic evidence for a causal role of obesity in diabetic kidney disease. Diabetes.

[10] Després J-P (2001). Health consequences of visceral obesity. Ann. Med..

[11] Kang SH, Cho KH, Park JW, Yoon KW, Do JY (2015). Association of visceral fat area with chronic kidney disease and metabolic syndrome risk in the general population: analysis using multi-frequency bioimpedance. Kidney Blood Press. Res..

[12] Sarathy H (2016). Abdominal obesity, race and chronic kidney disease in young adults: results from NHANES 1999–2010. PLoS ONE.

[13] Browning LM, Hsieh SD, Ashwell M (2010). A systematic review of waist-to-height ratio as a screening tool for the prediction of cardiovascular disease and diabetes: 0·5 could be a suitable global boundary value. Nutr. Res. Rev..

[14] Ashwell M, Hsieh SD (2005). Six reasons why the waist-to-height ratio is a rapid and effective global indicator for health risks of obesity and how its use could simplify the international public health message on obesity. Int. J. Food Sci. Nutr..

[15] Ashwell M, Lejeune S (1996). Ratio of waist circumference to height may be better indicator of need for weight management. BMJ.

[16] Ashwell M, Gunn P, Gibson S (2012). Waist-to-height ratio is a better screening tool than waist circumference and BMI for adult cardiometabolic risk factors: systematic review and meta-analysis. Obes. Rev. J..

[17] Krakauer NY, Krakauer JC (2012). A new body shape index predicts mortality hazard independently of body mass index. PLoS ONE.

[18] Hsieh SD, Yoshinaga H (1995). Abdominal fat distribution and coronary heart disease risk factors in men-waist/height ratio as a simple and useful predictor. Int. J. Obes. Relat. Metab. Disord. J..

[19] Kang DO (2019). prognostic impact of low skeletal muscle mass on major adverse cardiovascular events in coronary artery disease: a propensity score-matched analysis of a single center all-comer cohort. J. Clin. Med..

[20] Groop PH (2018). Excess mortality in patients with type 1 diabetes without albuminuria-separating the contribution of early and late risks. Diabetes Care.

[21] Spoto B, Pisano A, Zoccali C (2016). Insulin resistance in chronic kidney disease: a systematic review. Am. J. Physiol. Ren. Physiol..

[22] Cleasby ME, Jamieson PM, Atherton PJ (2016). Insulin resistance and sarcopenia: mechanistic links between common co-morbidities. J. Endocrinol..

[23] DeFronzo RA (1981). Insulin resistance in uremia. J. Clin. Invest..

[24] Lo K (2019). Prospective associations of waist-to-height ratio with cardiovascular events in postmenopausal women: results from the women’s health initiative. Diabetes Care.

[25] Groop P-H (2009). The presence and severity of chronic kidney disease predicts all-cause mortality in type 1 diabetes. Diabetes.

[26] Stenvinkel P, Carrero JJ, von Walden F, Ikizler TA, Nader GA (2016). Muscle wasting in end-stage renal disease promulgates premature death: established, emerging and potential novel treatment strategies. Nephrol. Dial. Transplant..

[27] Robinson KA, Baker LA, Graham-Brown MPM, Watson EL (2020). Skeletal muscle wasting in chronic kidney disease: the emerging role of microRNAs. Nephrol. Dial. Transplant..

[28] Biolo G (2015). Inverse relationship between ‘a body shape index’ (ABSI) and fat-free mass in women and men: Insights into mechanisms of sarcopenic obesity. Clin. Nutr. Edinb. Scotl..

[29] Gomez-Peralta F (2018). Relationship between ‘a body shape index (ABSI)’ and body composition in obese patients with type 2 diabetes. Diabetol. Metab. Syndr..

[30] Heo M, Faith MS, Pietrobelli A, Heymsfield SB (2012). Percentage of body fat cutoffs by sex, age, and race-ethnicity in the US adult population from NHANES 1999–2004. Am. J. Clin. Nutr..

[31] Frankenfield DC, Rowe WA, Cooney RN, Smith JS, Becker D (2001). Limits of body mass index to detect obesity and predict body composition. Nutr. Burbank Los Angel. Cty. Calif.

[32] Lee CMY, Huxley RR, Wildman RP, Woodward M (2008). Indices of abdominal obesity are better discriminators of cardiovascular risk factors than BMI: a meta-analysis. J. Clin. Epidemiol..

[33] Dobbelsteyn CJ, Joffres MR, MacLean DR, Flowerdew G (2001). A comparative evaluation of waist circumference, waist-to-hip ratio and body mass index as indicators of cardiovascular risk factors. The Canadian Heart Health Surveys. Int. J. Obes. Relat. Metab. Disord..

[34] World Health Organization. *Waist circumference and waist-hip ratio: report of a WHO expert consultation, Geneva, 8–11 December 2008*. (World Health Organization, Geneva, 2011).

[35] Meredith-Jones K, Haszard J, Stanger N, Taylor R (2018). Precision of DXA-derived visceral fat measurements in a large sample of adults of varying body size. Obes. Silver Spring Md..

[36] WHO Consultation on Obesity (1999: Geneva) & World Health Organization. Obesity : preventing and managing the global epidemic : report of a WHO consultation. *Obésité Prév. Prise En Charge Épidémie Mond. Rapp. Une Consult. OMS* (2000).

[37] Alberti KGMM, Zimmet P, Shaw J (2006). Metabolic syndrome: a new world-wide definition. A Consensus Statement from the International Diabetes Federation. Diabet. Med..

[38] Hainsworth DP (2019). risk factors for retinopathy in type 1 diabetes: the DCCT/EDIC study. Diabetes Care.

[39] R Core Team. *R: A Language and Environment for Statistical Computing*. (R Foundation for Statistical Computing, Vienna, 2018).

