# Supplementary material for: Waist-height ratio and waist are the best estimators of visceral fat in type 1 diabetes
Source: Sci Rep. 2020 Oct 29;10:18575. doi: 10.1038/s41598-020-75667-5 (PMC7596092; doi:10.1038/s41598-020-75667-5)
Supplement: Supplementary file 1 — Supplementary Information. [file 41598_2020_75667_MOESM1_ESM.docx]

**Full title**

Waist-height ratio and waist are the best estimators of visceral fat in type 1 diabetes

**Authors:**

Erika B Parente^†^, Stefan Mutter^†^, Valma Harjutsalo, Aila J Ahola, Carol Forsblom, Per-Henrik Groop^*^

^† shared first authorship, *correspondence author^

Table S1. Misclassification of body fat percentage by WC.

|  | WC < 94,80 | WC ≥ 94,80 | TOTAL |
| --- | --- | --- | --- |
| Normal body fat percentage (n, %) | 148 (24.5) | 24 (4.0) | 172 (28.5) |
| Excess body fat percentage (n, %) | 112 (18.6) | 319 (52.9) | 431 (71.5) |
| Number (%) of DXAs | 260 (43.1) | 343 (56.9) | 603 (100.00) |

The normal body fat percentage was considered: ≤ 25 for men and ≤ 30 for women. DXA: Dual-energy X-Ray Absorptiometry. Percentages are based on the total number of 603 scans. Waist circumference (WC) was considered normal if < 94cm for men and < 80cm for women.

Table S2. Misclassification of body fat percentage by WHR.

|  | WHR < 0.9,0.85 | WHR ≥ 0.9,0.85 | TOTAL |
| --- | --- | --- | --- |
| Normal body fat percentage (n, %) | 117 (19.4) | 55 (9.1) | 172 (28.5) |
| Excess body fat percentage (n, %) | 169 (28.0) | 262 (43.4) | 431 (71.5) |
| Number (%) of DXAs | 286 (47.4) | 317 (52.5) | 603 (100.00) |

The normal body fat percentage was considered: ≤ 25 for men and ≤ 30 for women. DXA: Dual-energy X-Ray Absorptiometry Percentages are based on the total number of 603 scans. Waist-hip ratio (WHR) was considered normal if <0.9 for men and <0.85 for women.

Table S3. Misclassification of body fat percentage by WHtR.

|  | WHtR < 0.5 | WHtR ≥ 0.5 | TOTAL |
| --- | --- | --- | --- |
| Normal body fat percentage (n, %) | 142 (23.5) | 30 (5.0) | 172 (28.5) |
| Excess body fat percentage (n, %) | 130 (21.6) | 301 (49.9) | 431 (71.5) |
| Number (%) of DXAs | 272 (45.1) | 331 (54.9) | 603 (100.00) |

The normal body fat percentage was considered: ≤ 25 for men and ≤ 30 for women. DXA: Dual-energy X-Ray Absorptiometry. Percentages are based on the total number of 603 scans. Waist-height ratio (WHtR) was considered normal if < 0.5 for both sexes.
